# Diversity and authentication of *Rubus* accessions revealed by complete plastid genome and rDNA sequences

**DOI:** 10.1080/23802359.2021.1911712

**Published:** 2021-04-20

**Authors:** Young Sang Park, Jee Young Park, Jung Hwa Kang, Wan Hee Lee, Tae-Jin Yang

**Affiliations:** a Department of Agriculture, Forestry and Bioresources, Plant Genomics and Breeding Institute, College of Agriculture and Life Sciences, Seoul National University, Seoul, Korea; b Hantaek Botanical Garden, Yongin, Korea

**Keywords:** *Rubus longisepalus*, *R. hirsutus*, chloroplast, rDNA, phylogenetic tree

## Abstract

Complete plastid genome (plastome) and ribosomal DNA (rDNA) sequences of three *Rubus* accessions (two *Rubus longisepalus* and one *R. hirsutus*) were newly assembled using Illumina whole-genome sequences. *Rubus longisepalus* Nakai and *R. longisepalus* var. tozawai, described as different varieties, have identical plastomes and rDNA sequences. The plastomes are 155,957 bp and 156,005 bp and the 45S rDNA transcription unit sizes are 5809 bp and 5811 bp in *R. longisepalus* and *R. hirsutus*, respectively. The 5S rDNA transcription unit is an identical 121 bp in three *Rubus* accessions. We developed three DNA markers to authenticate *R. longisepalus* and *R. hirsutus* based on plastome diversity. Phylogenomic analysis revealed that the *Rubus* species classified as two clades and *R. longisepalus*, *R. hirsutus*, and *R. chingii* are the most closely related species in clade 1.

## Introduction

The genus *Rubus* consists of about 500 species, for which the taxonomy remains unclear due to frequent hybridizations, polyploidization, and asexual reproduction (Alice and Campbell 1999; Wang et al. 2016; Hytönen et al. 2018). The genus has been divided into 12 subgenera (Focke 1910, 1914). However, this classification is not unanimously supported, and each subgenus has been reported to be non-monophyletic (Alice and Campbell 1999; Yang et al. 2012; Wang et al. 2016; Hummer et al. 2019). Even though previous studies contributed to current phylogenetic outline, short barcode regions such as internal transcribed spacer (ITS) and universal barcoding loci in the plastid genomes (plastome) have its own limitations (Li et al. 2015). Recently, nuclear genome and whole plastomes were used to analyze phylogenetic relationships among members of the genus *Rubus* and the chromosome scale genome assembly was released for *R. occidentalis* (VanBuren et al. 2016; Jibran et al. 2018; VanBuren et al. 2018; Hummer et al. 2019; Yang et al. 2021).

A super-barcoding approach using whole plastomes offers a solution to the limitations of using short barcoding regions to clearly distinguish inter- and intra-species diversity (Hollingsworth et al. 2009; Li et al. 2015). Since the plastome is inherited maternally in many plants, the absence of recombination preserves genome size, number of genes, and gene order in most plants (Palmer 1985; Wicke et al. 2011). However, sufficient variations are accumulated between species to allow estimation of their evolutionary path (Wolfe et al. 1987).

Nuclear ribosomal DNA (rDNA) exists in the plant nuclear genome in the form of thousands of tandem repeat arrays (Roa and Guerra 2012). Despite being part of the nuclear genome, its sequences are very conserved (Malinska et al. 2010). However, the internal transcribed sequences (ITS1 and ITS2) separating subunits of 45S rDNA (18S, 5.8S, and 28S) possess a meaningful level of variation among species (Álvarez and Wendel 2003). Whole-genome sequences produced by second- and third-generation sequencing platforms allow complete plastome and rDNA sequences to be assembled simultaneously in a time- and cost-effective manner (Kim et al. 2015a; Kim et al. 2015b). Comparison of plastomes and rDNA sequences have proved very useful for phylogenetic analysis and development of barcoding markers (Kim et al. 2017; Lee et al. 2019; Nguyen et al. 2020; Lee et al. 2021).

*Rubus longisepalus* Nakai, *R. longisepalus* var. *tozawai* (Nakai) T.B.Lee, are endemic to the Southern coasts and islands of the Korean Peninsula while *R. hirsutus* Thunb are distributed widely in Eastern Asia. *R. longisepalus* Nakai and *R. longisepalus* var. tozawai are regarded as distinct varieties with the common names ‘Macdo’ and ‘Geoje,’ respectively. *R. hirsutus* has a similar habitat and morphology as the two *R. longisepalus* varieties. Therefore, clear taxonomic identification and development of molecular markers are necessary for distinguishing these edible plant resources on the Korean Peninsula.

## Material and methods

### Plant materials and genome sequencing

Leaf samples of three *Rubus* accessions were provided from the Hantaek Botanical Garden, Gyeonggi-do, Republic of Korea. Each sample was ground into powder form using liquid nitrogen, and DNA was extracted using an Exgene Plant SV Midi Kit (Geneall Biotechnology, Seoul) following the manufacturer’s protocol. The extracted DNA was sequenced on the Illumina Miseq platform by Phyzen (www.phyzen.com, Seongnam, Gyeonggi-do). Approximately 1.3 Gbp paired-end sequence data were obtained for each of the three accessions.

### Assembly and annotation of plastomes and rDNAs

Plastomes and 45S rDNA sequences were assembled using the *de novo* assembly of low-coverage whole-genome sequencing (dnaLCW) method (Kim et al. 2015b). To summarize, raw reads were trimmed using the trimming tool in CLC Assembly and then assembled de novo using the CLC novo assembly tool (CLC Inc, Denmark). Only contigs with similarity to the reference plastid genome (*Rubus trifidus*, NC_046585.1) were extracted using MUMmer (Kurtz et al. 2004). Contigs structurally identical to the reference plastome were then extracted, and assembly of the three *Rubus* plastomes was completed through manual curation. The complete plastomes were annotated using GeSeq (https://chlorobox.mpimp-golm.mpg.de/geseq.html), with manual curation using artemis (Carver et al. 2012; Tillich et al. 2017). Finally, a gene map was drawn using OGDRAW (https://chlorobox.mpimp-golm.mpg.de/OGDraw.html) (Greiner et al. 2019). The 45S rDNA sequences were assembled in the same way. Contigs similar to the reference (*Sorbus commixta*, MN215997.1) were selected and curated manually. After assembly, each subunit (18S, ITS1, 5.8S, ITS2, 28S) was determined using RNAmmer followed by comparison with a reference (Lagesen et al. 2007). The 5S rDNA sequences were assembled using the reference mapping method. Reads were first mapped to the reference (*Arabidopsis thaliana*, AF330993.1), and then different positions were modified. Intergenic spacer regions (IGS) in 45S rDNA and 5S rDNA were characterized by extending the end position of the rDNA unit through read mapping. Extension of the IGS proceeded until the IGS sequence met the start position of the next rDNA subunit. Manual curation was then conducted to obtain complete rDNA repeats sequences.

### Polymorphism and marker development

The three completed chloroplast genomes and rDNA sequences were aligned using the MAFFT online version (Katoh et al. 2019). Plastome and rDNA variants were confirmed from the alignment results. Among the polymorphic regions, two single nucleotide polymorphisms (SNPs) and one insertion and deletion (InDel) region were selected for marker development. The two SNPs were developed into derived cleaved amplified polymorphic sequences (dCAPS) markers using dCAPS finder 2.0 (http://helix.wustl.edu/dcaps/) (Neff et al. 2002) and the InDel region was developed into a codominant marker. The three primer sets for these markers were validated in silico using NCBI primer blast (Ye et al. 2012) before adapting them to the three *Rubus* species (Table 1).

**Table 1.** Authentication markers and primers developed in this study.

Primer	Location	Product size (bp)	Recognition enzyme	Strand	Primer sequence
RubusdCAPS1	*psbA*	125 (RL)	*Mbo*I	F	CCAAGGTTAGCGCGGTTAAT
		148 (RH)		R	GGCCTGTAGTAGGTATCTGGAT
RubusdCAPS2	*atpI*	162 (RL)		F	AGGATTGGGGTTGGTTGAA
		137 (RH)	*Xho*I	R	GAAAATCATACAGTTACCTCCTCG
RubusInDel1	*trnS–trnG*	226 (RL)		F	GGGGCTTTTTAGTTTCACGGC
		278 (RH)		R	TGTGTCAAGAAACGACAGTTCC

*Mbo*I and *Xho*I were used for the dCAPS markers. F and R, forward strand and reverse strand, respectively. RL and RH, *R. longisepalus* and *R. hirsutus*, respectively.

### Phylogenetic analysis

A phylogenetic tree was reconstructed using coding sequences (CDSs) in the plastome. Sequences representing 11 additional species of the genus *Rubus* and three outgroup species also belonging to the family Rosaceae were obtained from NCBI GenBank (https://www.ncbi.nlm.nih.gov/genbank/). Only 74 CDSs common to the 16 species were extracted by FeatureExtract (Wernersson 2005). These sequences were concatenated into one contig. The 16 CDS contigs were aligned using PRANK with the translate option (Löytynoja 2014), and a phylogenetic tree was reconstructed using the maximum-likelihood method in MegaX with 1000 bootstrap replicates (Kumar et al. 2018).

## Results

### Characteristics of complete plastomes

**Figure 1.**
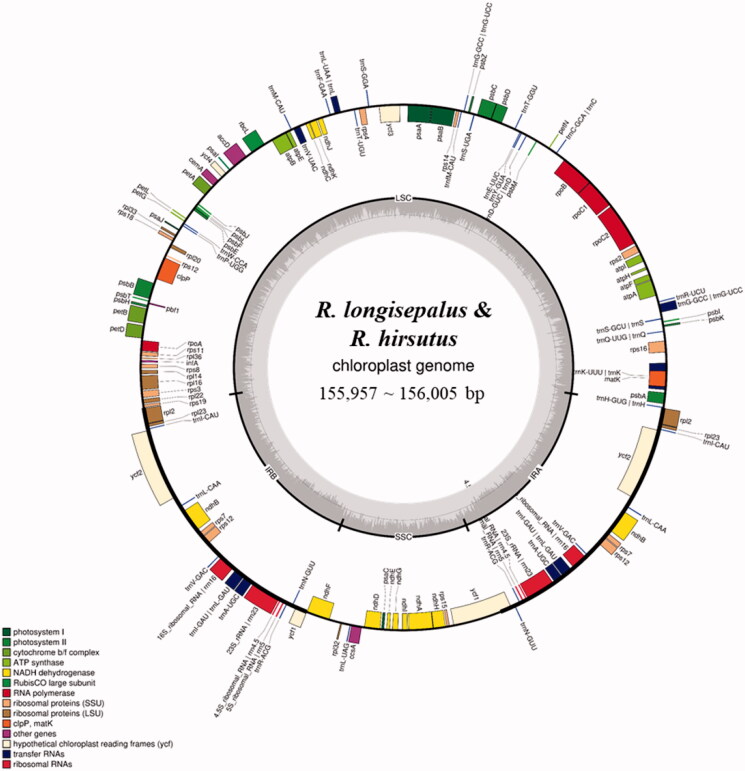
Chloroplast gene map of *R. longisepalus* and *R. hirsutus.* The total length of plastomes ranges from 155,957 to 156,005 bp.

Assembled plastomes have distinct quadripartite structures consisting of one long single copy (LSC), one short single copy (SSC), and two inverted repeats (IRb and IRa). *Rubus longisepalus* Nakai and *R. longisepalus* var. tozawai have completely identical plastomes. Both have a total length of 155,957 bp, with 85,633 bp of LSC, 18,766 bp of SSC, and 25,779 bp of IR. The *R. hirsutus* plastome has a total length of 156,005 bp, with 85,745 bp of LSC, 18,734 bp of SSC, and 25,763 bp of IR. Both species have the same gene content and order: 85 CDSs, 37 tRNAs, and 8 rRNAs (Table 2; Figure 1). Analysis of nucleotide variations between *R. longisepalus* and *R. hirsutus* revealed 1882 SNPs and 325 InDels.

**Table 2.** Information on newly assembled chloroplast genomes.

	Length (bp)				No. of genes			GenBank accession
Species	Total	LSC	IR	SSC	CDS	tRNA	rRNA	
*R. longisepalus*	155,957	85,633	25,779	18,766	85	37	8	MW436703
*R. hirsutus*	156,005	85,745	25,763	18,734	85	37	8	MW448480

### Marker development

**Figure 2.**
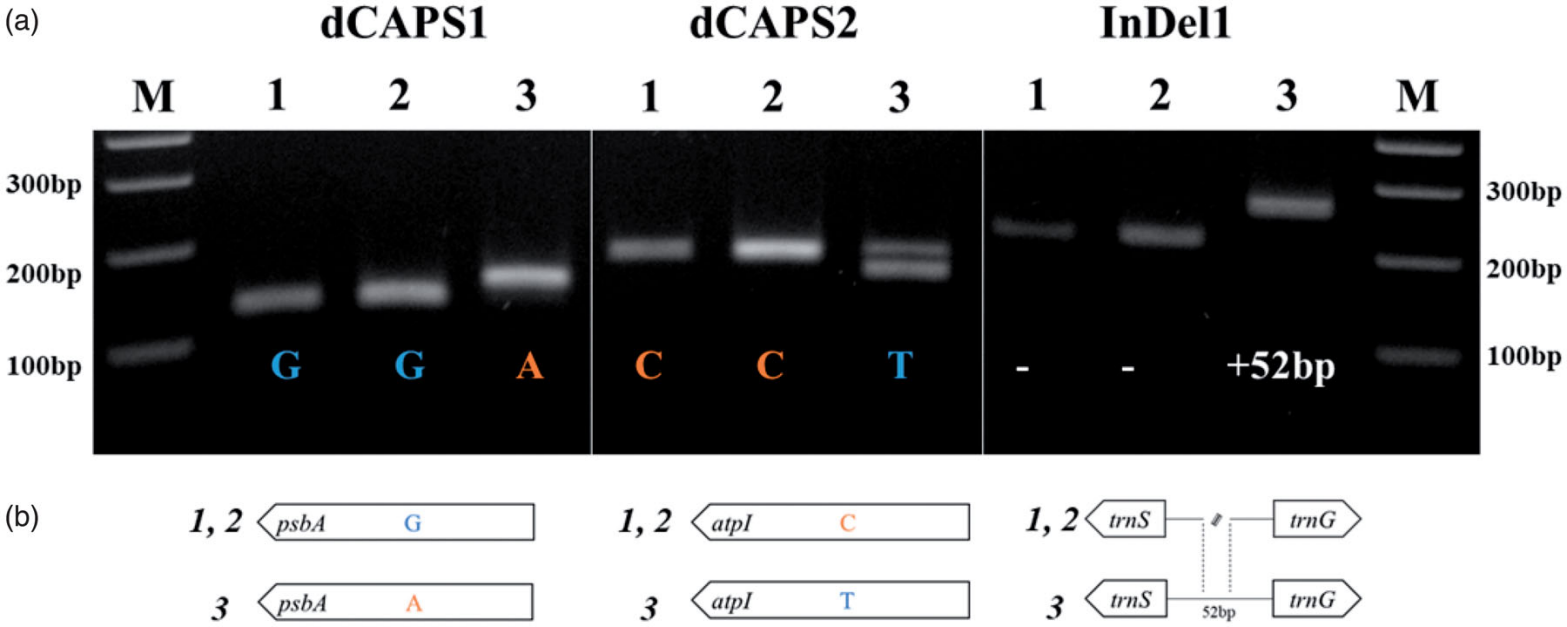
DNA marker validation and polymorphisms. (a) Agarose gel electrophoresis using three primer combinations. Detailed marker information including restriction enzymes and product sizes is provided in Table 1. M indicates 100 bp DNA ladder. 1, 2, and 3 indicate *R. longisepalus* Nakai, *R. longisepalus* var. *tozawai* and *R. hirsutus,* respectively. (b) Schematic diagram for the polymorphic sites between *R. longisepalus* and *R. hirsutus*.

We developed molecular markers based on the polymorphism between plastomes of *R. longisepalus* and *R. hirsutus*, and applied these to the three *Rubus* accessions. Sequence-based alignment of two dCAPS markers based on SNP regions and one codominant marker based on an InDel region confirmed their targets as polymorphic regions. All three markers could successfully distinguish *R. longisepalus* and *R. hirsutus* (Figure 2), validating the sequence assembly.

### Phylogenetic analysis

**Figure 3.**
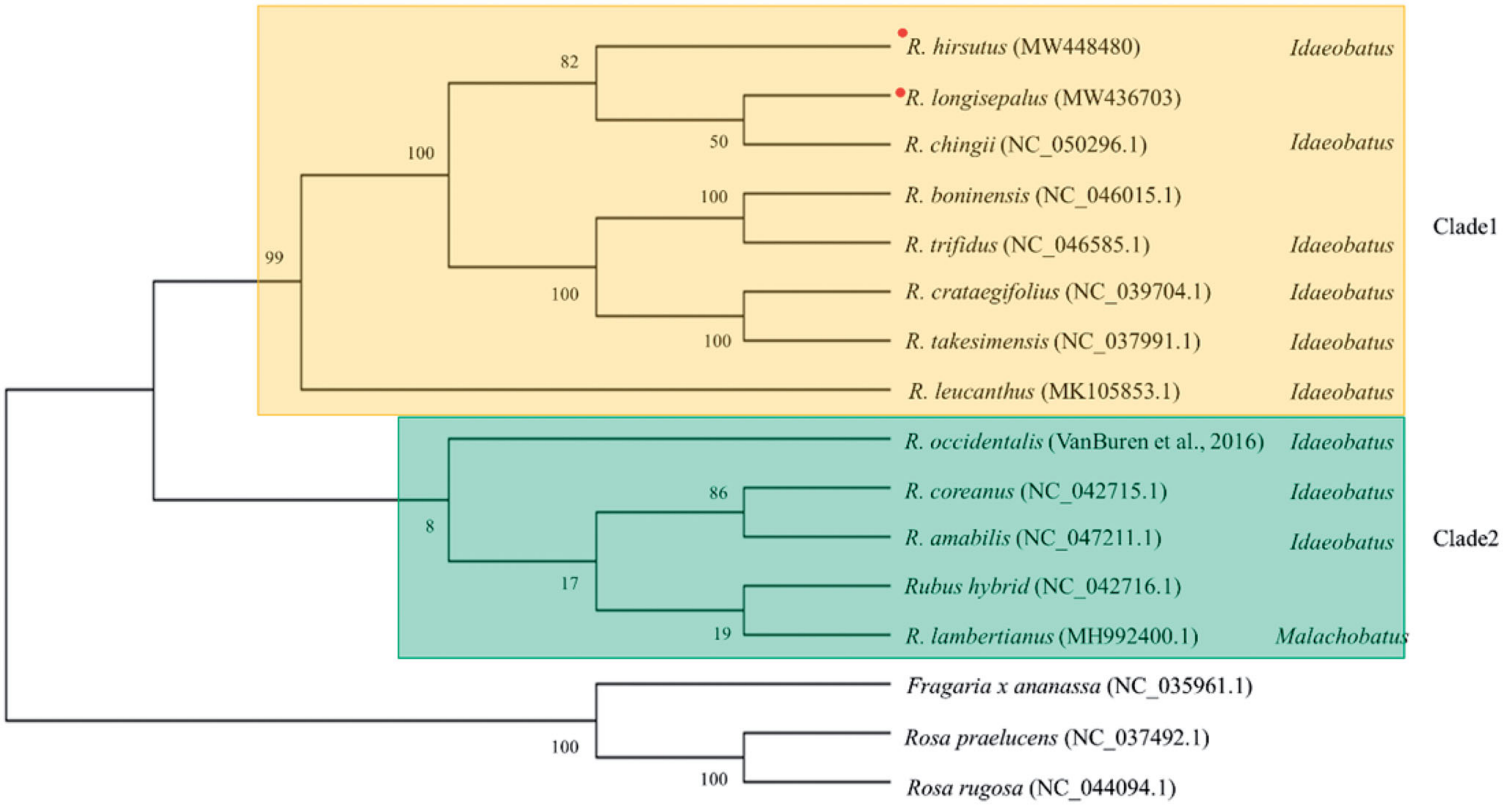
Phylogenetic tree of the genus *Rubus*. Concatenation of 74 common CDSs from 13 species of the genus *Rubus* was used to reconstruct a phylogenetic tree using the maximum-likelihood method in MegaX. Numbers at nodes are bootstrap values (as percentages) from 1000 replicates. Three additional species in the family Rosaceae were used as an outgroup. Species assembled in this study were marked with red circle.

To elucidate phylogenetic locations of *R. longisepalus* and *R. hirsutus*, plastomes of 11 additional species of the genus *Rubus* and three other species of the family Rosaceae were retrieved from NCBI GenBank. A total of 74 common CDSs were used to reconstruct and analyze a phylogenetic tree (Figure 3). Ten of the 13 *Rubus* species are classified into two subgenera in the GRIN database (https://npgsweb.ars-grin.gov/gringlobal/taxon/taxonomysearch): nine in the subgenus *Idaeobatus* and one in the subgenus *Malachobatus*. Meanwhile, our phylogenomic analysis classified the 13 *Rubus* species as two clades. Eight species including *R. longisepalus* and *R. hirsutus* fell into clade 1, with all species belonging to the monophyletic subgenus *Idaeobatus*, while the other five species belonged to clade 2, which is non-monophyletic and contains two subgenera. *R. lambertianus*, classified in subgenus *Malachobatus* based on GRIN database (https://npgsweb.ars-grin.gov/gringlobal/taxon/taxonomysearch), and three other *Rubus* species belonging to subgenus *Idaeobatus* were placed in clade 2. *R. longisepalus*, *R. hirsutus*, and *R. chingii* in clade 1 were the most closely related species among the 13 *Rubus* species studied.

### Nuclear rDNAs in R. longisepalus and R. hirsutus

**Figure 4.**
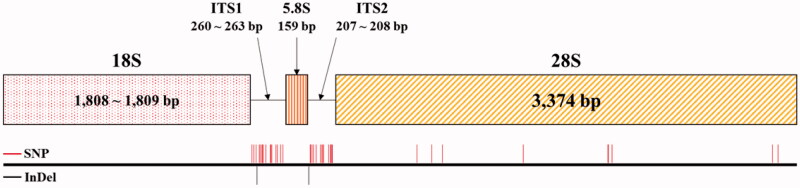
Structure and nucleotide variation between the 45S rDNAs of *R. longisepalus* and *R. hirsutus*. The diagram of 45S rDNA structure was represented with 18S, ITS1, 5.8S, ITS2, 26S rRNAs. Red and black lines denote SNP and InDel positions between *R. longisepalus* and *R. hirsutus*, respectively.

We assembled complete rDNA units including transcription units and inter genic spaces (IGS) for all three *Rubus* accessions. The 45S rDNA and 5S rDNA units were assembled independently as repeated array forms. The 45S rDNA unit contains a transcription unit of 5809–5811 bp spanning 10,093 bp to 10,630 bp including IGS. The 5S rDNA has a 121-bp transcription unit spanning 499 bp to 501 bp including IGS (Table 3; Figure 4). The transcription units in the 45S rDNA subunit are similar sizes in the two species, excluding ITS1 and ITS2, which are known to accumulate variations relatively fast. ITS1 and ITS2 of *R. hirsutus* are different from those of *R. longisepalus*. The 5S rDNA transcription unit sequences are the same among all three accessions. The IGS of 45S rDNA are different among all three accessions, while the IGS of 5S rDNA are the same in the two *R. longisepalus* accessions but differ from those of *R. hirsutus*.

**Table 3.** rDNA assembly information for *R. longisepalus* and *R. hirsutus*.

Kinds	Regions	*R. longisepalus* Nakai	*R. longisepalus* var. *tozawai*	*R. hirsutus*
45S rDNA	18S RNA	1808	1808	1808
	ITS1	260	260	263
	5.8S RNA	159	159	159
	ITS2	208	208	207
	28S RNA	3374	3374	3374
	IGS	4714	4821	4282
	GenBank accession	MW474728	MW474727	MW474729
5S rDNA	5S RNA	121		121
	IGS	380		378
	GenBank accession	MW474730		MW474731

## Discussion

Completion of three newly assembled *Rubus* plastomes and rDNA sequences allowed us to identify their polymorphisms and phylogenetic relationships. Two accessions of *R. longisepalus*, known to represent the same species but classified as different varieties, have identical plastomes and rDNA sequences. Despite large variations between *R. longisepalus* and *R. hirsutus*, they are the most closely related species among the 13 species of the genus *Rubus* studied. The majority of species in the genus *Rubus* belong to the subgenus *Idaeobatus*, with only one species classified as subgenus *Malachobatus*. Since most of the branches reconstructed in this study correspond with those obtained in previous studies, we conclude that the overall topology of our phylogenetic tree is reliable (Yang and Pak 2006; Yang et al. 2012; Wang et al. 2016; Hummer et al. 2019; Wang et al. 2020; Yang et al. 2021). The genome data and barcode markers developed in this study provide a basis for unveiling the phylogenetic relationships of species of the genus *Rubus* worldwide.

## Data Availability

The data that support the findings in this study are available at NCBI GenBank (https://www.ncbi.nlm.nih.gov/genbank/). Plastome accession number are *R. longisepalus* (MW436703), *R. hirsutus* (MW448480). Accession number of 45S rDNA with IGS are *R. longisepalus* Nakai (MW474728), *R. longisepalus* var. *tozawai* (MW474727) and *R. hirsutus* (MW474729). Accession number of 5S rDNA with IGS are *R. longisepalus* Nakai (MW474730), *R. longisepalus* var. *tozawai* (MW474730) and *R. hirsutus* (MW474731). SRA accession number are *R. longisepalus* Nakai (SRR14026745), *R. longisepalus* var. *tozawai* (SRR14027373) and *R. hirsutus* (SRR14038231) under BioProject accession (PRJNA716145).
